# Cecal volvulus complicated by evisceration case report

**DOI:** 10.1093/jscr/rjaa562

**Published:** 2021-01-18

**Authors:** Kylie Johnson, Ben Williams, Eric Steen

**Affiliations:** Lewis Gale Medical Center, Edward Via College of Osteopathic Medicine, Blacksburg, VA 24060, USA; Lewis Gale Medical Center, Edward Via College of Osteopathic Medicine, Blacksburg, VA 24060, USA; Lewis Gale Medical Center, Edward Via College of Osteopathic Medicine, Blacksburg, VA 24060, USA

**Keywords:** Colorectal Surgery

## Abstract

This case of bowel obstruction with multiple postoperative complications provides unique insight into the challenges faced by providers caring for intellectually disabled patients with acute surgical abdominal pathology and poor compliance. In this case, the component separation was utilized as a method of facilitated wound closure and compliance in a postoperative course highlighted by both dehiscence and wound infection. The patient, only able to communicate the presence of abdominal pain due to his disability, was surgically managed for a bowel obstruction secondary to a cecal volvulus. The difficulty in initial communication and patient noncompliance help illustrate the individualized care these patients require. This report will demonstrate both the challenges present in the management of intellectually disabled patients with abdominal wounds, as well as the use of component separation in providing both initial wound closure and continued wound integrity with the goal of reducing postoperative complications in patients with decreased compliance.

## INTRODUCTION

Component separation is an alternative technique of midline abdominal wall closure that ‘enlarges the circumference of the abdomen by moving muscle layers to bridge the fascial defects’ [1]. In the technique used for our patient, the anterior abdominal fascia is separated bilaterally from the rectus abdominis muscle and external oblique aponeurosis from the costal margin to the anterior superior iliac spine. This creates subcutaneous skin flaps allowing the approximation of midline abdominal wounds. Advantages of this ‘technique are that it restores functional and structural integrity of the abdominal wall, provides stable soft tissue coverage, and optimizes aesthetic appearance’ [1]. Consequently, newly created skin flaps can suffer from compromised blood flow resulting in skin ischemia. In addition, ‘widespread elevation of skin flaps in open CS creates a large subcutaneous space, which increases the risk for seroma formation, wound infection, and wound dehiscence’ [2]. To reduce the incidence of these complications, drains and/or mesh may be placed prior to wound closure.

## CASE REPORT

**Figure 1:**
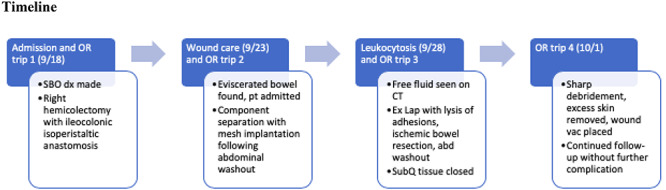
Hospital course and operative management.

A 50-year-old Caucasian male with autism presented with 1week of abdominal pain and vomiting progressing to obstipation. The patient’s limited communication skills led to a delayed diagnosis of a complete bowel obstruction. The patient underwent exploratory laparotomy which revealed extremely dilated loops of small bowel secondary to cecal volvulus, leading to a right hemicolectomy. He was discharged with instruction to return to the wound clinic for midline abdominal wound care. On return to the wound clinic, the patient’s abdominal wound was found to have dehiscence with bowel evisceration. This complication arose as the patient had difficulty understanding postoperative instruction to refrain from using his abdominal muscles. The subsequent OR trip consisted of component separation along with mesh implantation to allow for decreased tension on the wound hoping for complete closure and healing. A full timeline of his hospital course is referenced in Fig. 1.

### Patient info

The patient was a 50-year-old intellectually disabled male who arrived at the hospital as a transfer from an outside facility for a diagnosed small bowel obstruction. Prior to his transfer, the patient had nonoperative management of suspected partial bowel obstruction with gastric decompression, analgesia, nausea control and bowel rest. This yielded a resolution of his symptoms for 1 day, but the patient faced a rapid return of his symptoms, after which he was transferred to a higher level of care for operative management. While his disability significantly limited his communication with those involved, he was able to relay the presence of periumbilical abdominal pain with failure to pass flatus or stool.

The patient was autistic and legally blind. His past medical history consists of gout and hypertension. Past surgical history was significant for fracture repair of the right upper and lower extremities leading to gross deformity and negative for any abdominal surgeries. Family history consisted of hypertension and diabetes mellitus. The patient denied alcohol, tobacco, or illicit drug use. He was taking Thorazine, Lopressor and Allopurinol and had no known drug allergies.

### Clinical findings

The patient was an obese male in mild distress. He presented with a blood pressure of 121/72 mmHg, heart rate of 109 bpm, respiratory rate of 18 breaths/min, the temperature of 97.9 F and oxygen saturation of 96% on room air. Examination showed moist mucous membranes, no jugular venous distension and no peripheral edema. No rashes or abnormal bruising were noted. Heart sounds were regular in rate and rhythm. Lungs were clear to auscultation bilaterally. The abdomen was soft and distended with faint bowel sounds. Palpation elicited tenderness in the periumbilical region with no rebound or guarding.

## TIMELINE

### Diagnostic assessment

On presentation, CBC revealed a white count of 14 000/mm^3^, hemoglobin of 13.7 g/dl, and platelet count of 323 000/mm^3^. BMP was within normal limits. Differential diagnosis included small bowel obstruction, infectious gastroenteritis, ischemic bowel, peptic ulcer disease, appendicitis, cholecystitis and diverticulitis. CT of the abdomen and pelvis showed a high-grade small bowel obstruction with no pneumatosis. A favorable prognosis was anticipated with urgent surgical intervention.

### Therapeutic interventions

Following his transfer, a nasogastric tube was placed, antiemetics and analgesics were given, and an exploratory laparotomy was performed. His operative course began with a right hemicolectomy following the discovery of cecal volvulus. Pathology confirmed the presence of an incidental low-grade appendiceal mucinous neoplasm, which was postulated to be a contributing factor for the volvulus. Following an uncomplicated discharge, he returned for wound care with wound dehiscence and evisceration that was mistaken for subcutaneous fat at an outside facility 3 days prior. The patient was scheduled to return to the OR for wound closure and abdominal washout, with wound tension necessitating a component separation. To reduce the incidence of seroma formation, four 19-French Blake drains were placed in the subcutaneous spaces created. A biologic mesh implant was embedded to further enforce the wound and prevent an incisional ventral hernia from developing postoperatively. An additional utility in using biological mesh grafts is due to their ability to ‘provide a collagen and other extracellular matrix scaffold in which the host fibroblasts can create angiogenesis and create new collagen. The non-synthetic nature of these products allows them to be more resistant to infection’ [3]. The abdomen was then closed primarily.

### Follow-up and outcomes

Due to limited risk factors for surgical site infection, uncomplicated recovery was expected after the component separation given the decreased tension and reinforcement of the abdominal wound. While wound integrity was achieved, the patient developed leukocytosis 7 days following the procedure, as well as wound cultures positive for *Pseudomonas aeruginosa* and methicillin-resistant *Staphylococcus aureus*. The patient was placed on Vancomycin and Zosyn, empirically, and was taken back to the OR. Exploratory laparotomy was performed with lysis of adhesions, abdominal washout, and inspection of presumed perforation of the bowel. While no obvious perforation was found, a small segment of the ischemic bowel was discovered and resected; the subcutaneous tissue was closed, and the wound was left open. Two days later, a final operative trip was made for sharp debridement, excess skin removal, and wound vac placement, after which the patient had uncomplicated wound care follow-up. After complete healing of the wound, the patient underwent a split-thickness skin graft with no complications.

## DISCUSSION

Multiple aspects of this case speak to its strength in providing a clinical view of the utility of component separation in complicated wound closure. This case provided both a detailed view of the postoperative complications frequently encountered in those who are intellectually disabled, as well as a unique approach in the management of correcting these complications. While suspected bowel ischemia necessitated additional operations, the use of component separation allowed for maintained wound closure prior to the ischemic bowel and facilitated closure after resection of the ischemic bowel. Following final closure, the patient did not experience any further complications of dehiscence, despite his continued poor compliance with typical wound care. Longer follow-up to observe the long-term consequences of utilizing component separations, particularly the incidence of herniation postoperatively, would need to be assessed, given the high incidence following the use of component separation in other abdominal wall surgeries and repairs [4].

In the review of current literature, individuals with an intellectual disability provide multiple challenges in both initial and continued care. Data reviewing those intellectually disabled with acute surgical abdominal pathology supports the difficulty in initial diagnosis, as well as in the continued care of these individuals. As a result, there is an increased risk of unfavorable outcomes, such as postoperative acute renal failure, pneumonia, bleeding, septicemia and overall complications [5, 6]. This theme was also seen in the patient of this case report, who had a delayed diagnosis of wound evisceration, which is believed to have contributed to the complications faced with his ischemic bowel. In light of these increased risks given our patient’s disability, we could have considered component separation initially due to the large abdominal incision and the patient’s inability to understand postoperative instructions.

The patient in this study was not only a good candidate due to his poor compliance but also due to the nature of his wound. As he had a large ventral wound, the utilization of component separation was especially useful in this situation, as the technique is generally most often used in those with large, contaminated wounds or recurrent ventral hernia repairs [7]. Additionally, component separation allowed for appropriate fascial closure, which has proved to be instrumental in both protection from infection and dehiscence and in improvement of functional status. This is achieved as ‘reloading muscle at an appropriate tension, as opposed to a tension-free repair, leads to improved muscle mass and the generation of force’ [8].

In review, the intellectually disabled patient presents a unique challenge in the context of acute abdominal pathology and the need for wound closure. As is illustrated by both this report and the supporting data, these patients have significant difficulties with both diagnosis and the maintenance of wound integrity. In those patients who present an as high risk for wound dehiscence from poor compliance and large wound size, component separation can be used to provide primary closure as well as to maintain wound closure with the goal of reducing the rates of wound dehiscence and complications that occur with it.
